# 3D printing lunate prosthesis for stage IIIc Kienböck’s disease: a case report

**DOI:** 10.1007/s00402-017-2854-0

**Published:** 2017-12-12

**Authors:** Mei-ming Xie, Kang-lai Tang, Chen-song Yuan

**Affiliations:** Third Military Medical University Southwest Hospital, Chongqing, China

**Keywords:** 3D printing technology, Kienböck’s disease, Wrist, Avascular necrosis

## Abstract

Stage IIIc Kienböck’s disease is a clinical challenge to treat collapse of the lunate bone. A new reconstructive surgery was described in one patient using 3D printing lunate prosthesis. The prosthesis shape was designed by tomographic image processing and segmentation using technology compared with the intact side matched by mirror symmetry and 3D post-processing technologies. The patient recovered nearly full range of motion of the wrist after 12 months. The visual analog scale scores and Cooney scores were 2 points and 91 points. We demonstrated that an anatomical reconstruction to Kienböck’s Disease is possible using 3D printing lunate prosthesis.

## Introduction

The Kienböck’s disease is caused by avascular necrosis of the lunate bone [1]. According to the modified Lichtman radiologic classification, the disease is divided into four stages. Stage IIIc Kienböck’s disease is a clinical challenge to treat collapse of the lunate bone. It is a complete coronal plane split regardless of the lunate and wrist morphology [2, 3].

Various methods have been reported to stage IIIc, including transplant of pedicled pisiform bone, radial resection, radioscapholunate arthrodesis, scapho-trapezio-trapezoid arthrodesis and lunate bone replacement, etc [4–8]. Spies et al. and Unglaub et al. [9, 10] adopted proximal carpal row carpectomy and partial fusion of the wrist to obtain excellent clinical results. Bone transplantation cannot originally repair lunate shape and location. Arthrodesis can decrease the load and increase bone fusion, however, prevent the movement of the lunate between the distal rows of the carpal bone. So the restoration of anatomical structure and activity of lunate is a clinical problem.

With the development of three-dimensional (3D) printing, the technology was used to design and create complex replacement for bone structures [11–13]. If the cartilage of the radius and capitate were intact, by the way, in IIIc after Lichtman, it is untypical, so the prosthesis replacement surgery is an alternative option. The experimental case is the first report of 3D printing prosthesis which applied to stage IIIc Kienböck’s disease. The purpose was to present the new treatment method and clinical outcome.

## Case presentation

A 41-year-old visited to our hospital because of wrist pain for more than 2 years after an accidental fall. The patient felt wrist swelling, chronic pain, fatigue, limited activity in immoderate activity, unable to adhere to heavy manual jobs. The region of the lunate has a tenderness when palpated. Wrist was limited in each direction, especially in wrist dorsal extension. Plain radiographs and MRI showed that the lunate density was uneven and the height and width of lunate were collapsed when compared with the intact wrist (Fig. 4a–d). This study was approved by the Ethics Committee of the Southwest Hospital Affiliated with the Third Military Medical University. Informed consent was obtained from the patient. Preoperative MRI and X-rays showed that the lunate density was uneven and the height and width of lunate were collapsed.

**Fig. 1:**
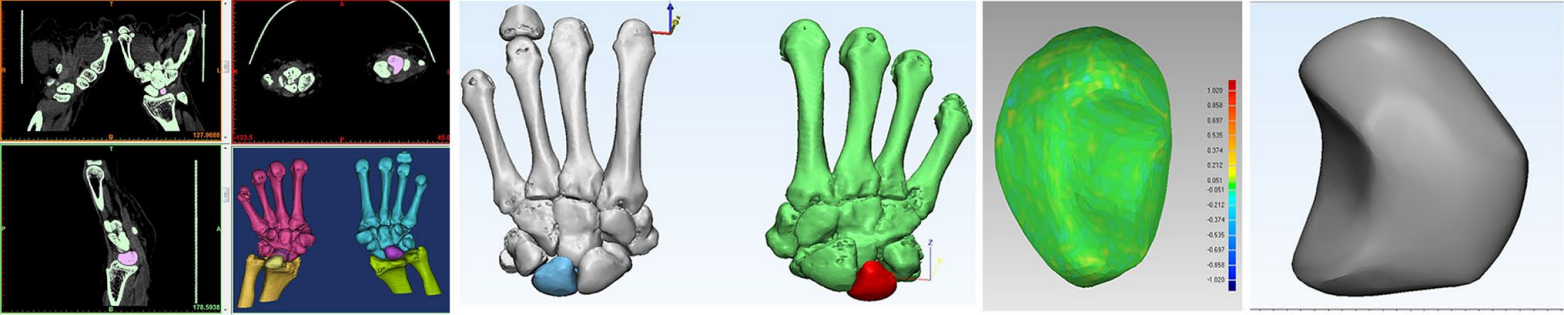
The necrotic lunate were reconstructed using reverse repairing technology to provide the non-defect data

The 3D printing technology applied to design and product lunate prosthesis. The complete data of the affected lunate were acquired by tomographic image processing and segmentation using 3D CT post-processing technology, and the intact 3D data of the affected side were obtained by reconstruction and matching based on mirror technology and data registration technology. The serious data defects of the necrotic lunate were repaired using reverse repairing technology to provide the non-defect data for lunate reconstruction (Fig. 1).

**Fig. 2:**
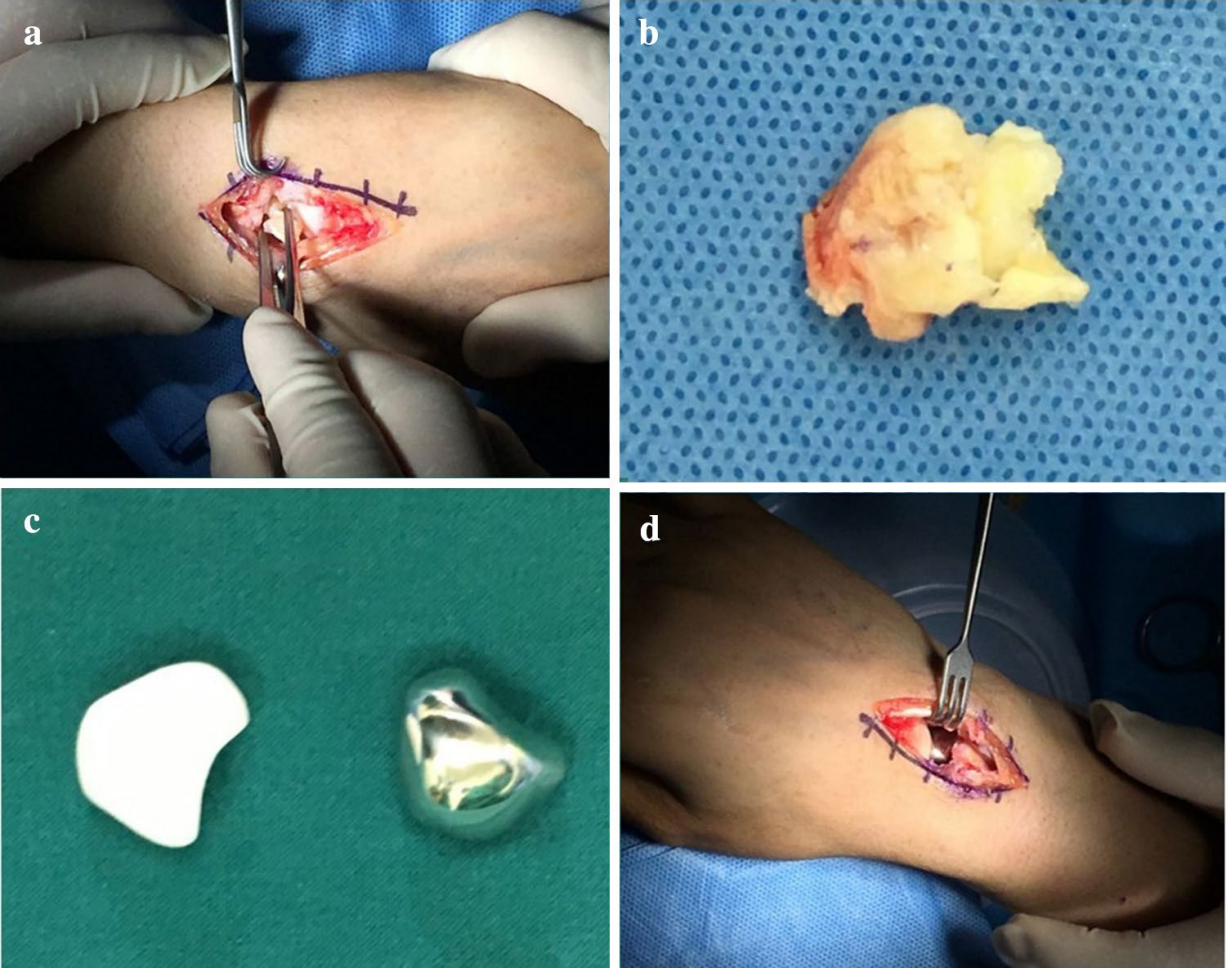
The necrotic lunate was resected (**a, b**); the 3D printing prosthesis was placed in the original anatomic position of the lunate (**c, d**)

**Fig. 3:**
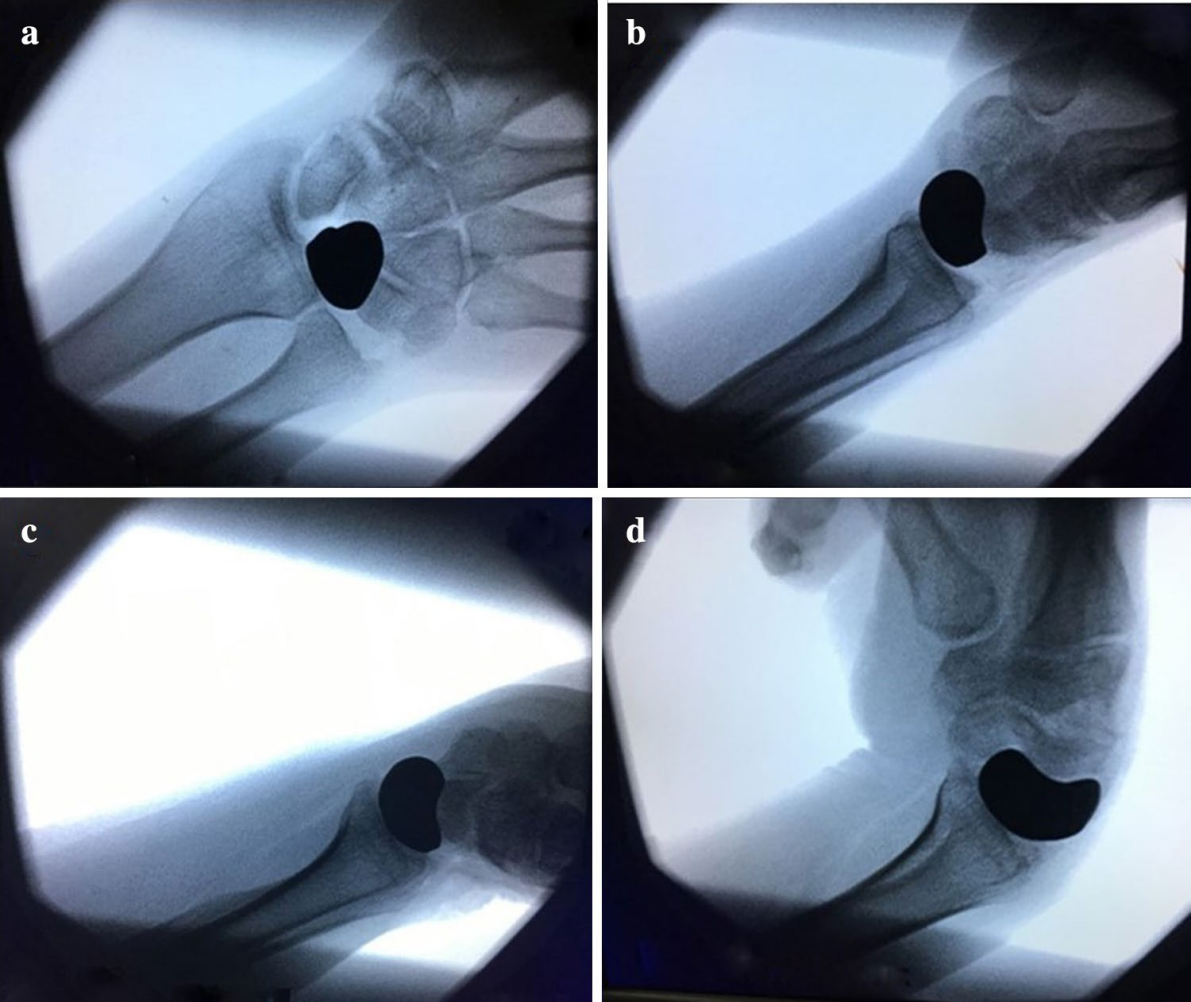
The prosthesis was check to be not dislocated during dorsal extension, palmar flexion, radial deviation and ulnar deviation of the wrist (**a**–**d**)

The new surgical technique was performed in a supine position under combined spinal epidural analgesia. First, the necrotic lunate was exposed with the dorsal skin incision and the lunate bone was resected. The cartilages of capitate and radius were smooth without the signs of osteoarthritis. The necrotic or inflammatory tissues were removed (Fig. 2a, b). The polyethylene prosthesis tested good matching performance of the 3D printing model. The 3D printing prosthesis was placed in the original anatomic position of the lunate (Fig. 2c, d). The prosthesis was check to be not dislocated during dorsal extension, palmar flexion, radial deviation and ulnar deviation of the wrist (Fig. 3). The dorsal articular capsule was sutured. The wrist was fixed with a plaster in the functional position for 4 weeks, and functional exercises of dorsiflexion and flexion to improve the range of wrist motion.

**Fig. 4:**
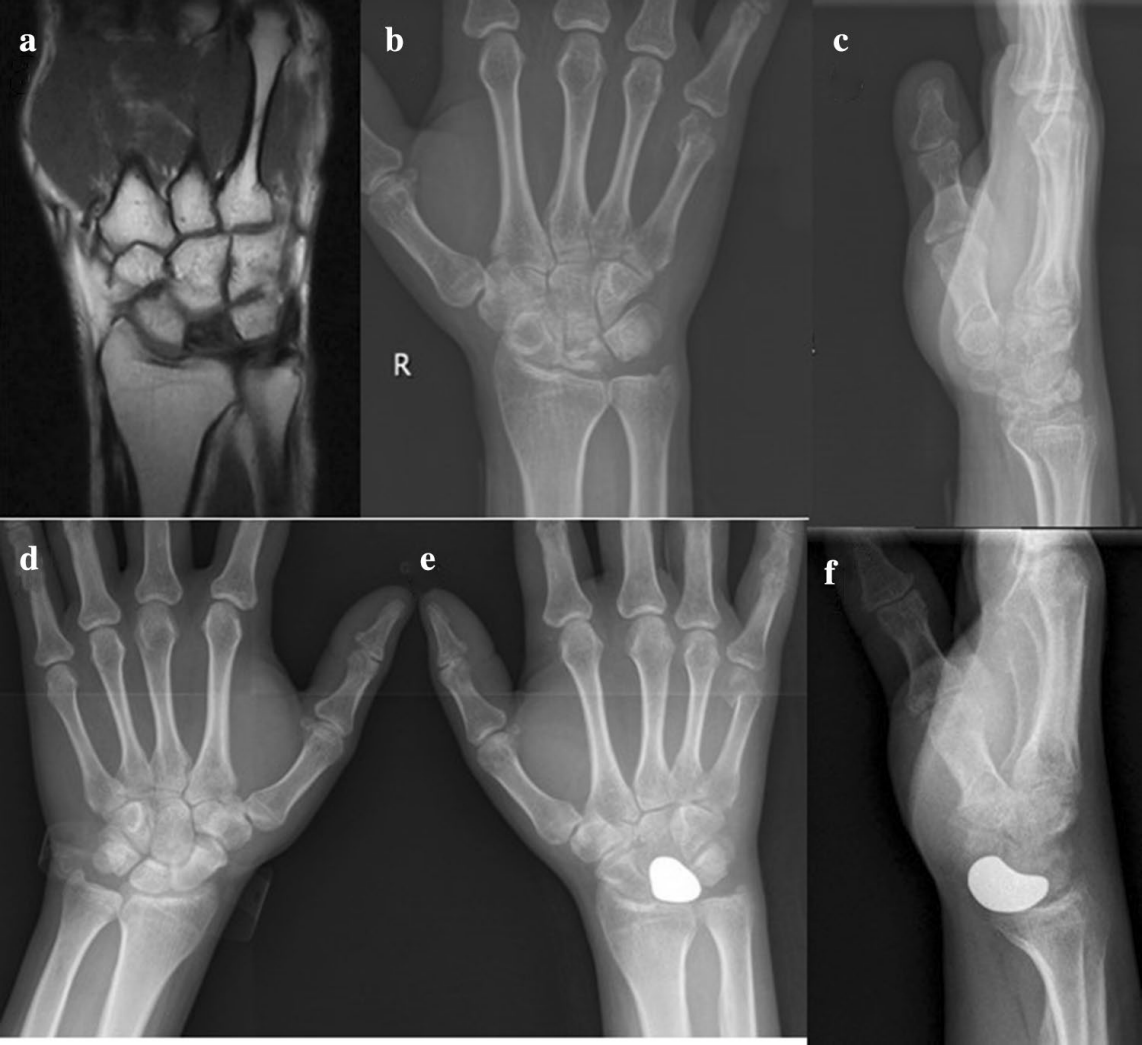
Preoperative MRI and X-rays showed that the lunate density was uneven and the height and width of lunate were collapsed (**a**–**c**); the intact wrist X-ray (**d**); the 3D printing lunate prosthesis was placed in the original anatomic position (**e, f**)

The patient was able to use her wrist with mild pain during sport activities after 12 months. Weakness and numbness of the wrist were not observed. Nearly full range of motion and grasp force of the wrist was obtained (Table 1). Degenerative arthritis and prosthetic dislocation on plain radiographs were not showed. The 3D printing lunate prosthesis was placed in the original anatomic position (Fig. 4e, f).The visual analog scale scores and Cooney scores were 2 points and 91 points.

**Table 1:** Comparison of wrist ROM and grasp force

	The affected wrist		The intact wrist
	Before surgery	At the last visit	
Dorsal extension (°)	30.7	54.2	56.3
Palmar flexion (°)	28.5	50.5	51.8
Ulnar deviation (°)	10.2	23.8	25.0
Radial deviation (°)	8.6	16.4	18.0
Grasp force (kg)	20.4	36.2	39.6

## Discussion

Stage IIIc Kienböck’s disease is a clinical challenge to anatomical reconstruction [14].The common treatment measure is carpal bone arthrodesis because of decreasing the load on the lunate bone, however, it might reduce wrist mobility [15]. If the cartilage of the radius and capitate were intact in stage IIIc and the surgeon maximum reserve articular cartilage and joint activity, the matched prosthesis was designed to apply in the reconstruction.So we attempt to reconstruct the original model of lunate prosthetic through 3D printing technology.

Actually, most of medical applications of 3D printing technology has been prosthesis [16]. 3D printing technology offers a new option to fabricate complex shape and structure of lunate [17, 18]. The accurate lunate imaging data were segmented and 3D reconstruct on the basis of CT and MRI. The intact lunate model was matched by a comparison of the healthy and affected sides using mirror symmetry and multidimensional computer reconstruction technology. It simulated the virtual surgical procedure to certify the operability of lunate prosthesis implantation. The 3D printing technology has allowed orthopedists to design and manufacture anatomically matched implants used in surgeries [19]. In the surgery, the 3D printing lunate returned to its original shape and location. It achieves a tight 3D match between the three carpal bones, respectively. Fixation stability was certified by rotating the wrist. The lunate implant position was checked using C-arm fluoroscopy. The prosthetic interface was smooth. It showed that the prosthesis provided a support to carpus after excision of the lunate bone and avoided adjacent osteoarthritis.

There are some reports for lunate replacement, including vitallium, acrylic materials or pyrocarbon lunate arthroplasty [20–22]. However, lunate replacement have not been popular because of the congruity and inherent instability [2]. The SL and LT ligaments were cut off and did not repaired after the prosthesis implantation. The stability of lunate prosthesis was primarily maintained by the surrounding bony structures. To prevent the dislocation and prolapse, the articular capsule was closely repaired and sutured. The plaster was fixed in the wrist to strength the repair of the articular capsule and the soft tissues and the wrist moved after 4 weeks to prevent the prosthesis dislocation. Compared with previous reports about lunate implantation, the 3D printing prosthesis shows the preservation of adjacent congruity. We believe 3D printing lunate prosthesis provides a reliable option to stage IIIc Kienböck’s disease by preserving lunate anatomy and the mobility of the wrist. The individualized 3D-printing lunate prosthesis might be designed according to the variation of lunate functional anatomy and the Wolf Law by composite computer information technologies in the future.

The interesting result is that an anatomical reconstruction to Kienböck’s disease is possible using 3D printing lunate prosthesis.
